# Polarization control of THz emission using spin-reorientation transition in spintronic heterostructure

**DOI:** 10.1038/s41598-020-80781-5

**Published:** 2021-01-12

**Authors:** Dinar Khusyainov, Sergei Ovcharenko, Mikhail Gaponov, Arseniy Buryakov, Alexey Klimov, Nicolas Tiercelin, Philippe Pernod, Vadim Nozdrin, Elena Mishina, Alexander Sigov, Vladimir Preobrazhensky

**Affiliations:** 1 MIREA - Russian Technological University, Moscow, Russia 119454; 2 Univ. Lille, CNRS, Centrale Lille, Univ. Polytechnique Hauts-de-France, UMR 8520 -IEMN, 59000 Lille, France; 3 Prokhorov General Physics Institute of RAS, Moscow, Russia 119991

**Keywords:** Optical spectroscopy, Magnetic properties and materials, Structural properties, Phase transitions and critical phenomena, Spintronics, Surfaces, interfaces and thin films, Optoelectronic devices and components, Ultrafast lasers, Magneto-optics, Terahertz optics

## Abstract

Polarization of electromagnetic waves plays an extremely important role in interaction of radiation with matter. In particular, interaction of polarized waves with ordered matter strongly depends on orientation and symmetry of vibrations of chemical bonds in crystals. In quantum technologies, the polarization of photons is considered as a “degree of freedom”, which is one of the main parameters that ensure efficient quantum computing. However, even for visible light, polarization control is in most cases separated from light emission. In this paper, we report on a new type of polarization control, implemented directly in a spintronic terahertz emitter. The principle of control, realized by a weak magnetic field at room temperature, is based on a spin-reorientation transition (SRT) in an intermetallic heterostructure TbCo_2_/FeCo with uniaxial in-plane magnetic anisotropy. SRT is implemented under magnetic field of variable strength but of a fixed direction, orthogonal to the easy magnetization axis. Variation of the magnetic field strength in the angular (canted) phase of the SRT causes magnetization rotation without changing its magnitude. The charge current excited by the spin-to-charge conversion is orthogonal to the magnetization. As a result, THz polarization rotates synchronously with magnetization when magnetic field strength changes. Importantly, the radiation intensity does not change in this case. Control of polarization by SRT is applicable regardless of the spintronic mechanism of the THz emission, provided that the polarization direction is determined by the magnetic moment orientation. The results obtained open the prospect for the development of the SRT approach for THz emission control.

## Introduction

Generation of terahertz (THz) radiation is of great technological importance for many current and future applications, such as non-destructive diagnostics, ultra-fast computing, wireless communications^1–4^, as well as direct control of the materials order parameters^5^.

During the last decade enormous work has been done to verify materials and mechanisms providing THz radiation with required characteristics. Among THz-materials are semiconductors ZnTe^6^, GaP^7^, GaSe^8^, GaAs^9^, organic crystals^10^, metals^11,12^, in which either optical rectification or photocurrents induced by a short optical pulse are the sources of THz radiation. Recently, not only charge but spin has been taken into consideration in spintronic THz emitters (STE), where ultrafast spin photocurrent can convert into a transverse charge current. Using STE, new applications such as near-field THz microscope have been suggested^13,14^.

The conventional STE is a bilayer made of ferromagnetic and nonmagnetic metal thin films stack^15,16^. In a ferromagnetic layer, magnetization lies in the film plane. An optical femtosecond pump pulse incident on the ferromagnetic/nonmagnetic structure generates a nonequilibrium electronic state. Since in a ferromagnetic layer with uncompensated magnetization, the mobility of spin-up (majority) electrons is significantly higher than that of spin-down (minority) electrons, the current appearing along the normal to the stack surface is spin-polarized. The inverse spin-Hall effect (ISHE) transforms the out-of-plane spin current into the desired sub-picosecond in-plane charge current. The latter gives rise to the emission of a THz electromagnetic pulse. The spectral range of the emitted radiation in such materials is from 0.5 to 15 THz (two orders of the dynamical range magnitude) with signal amplitude compared with that of ZnTe or InP^15^. The magnetic field value controls magnetization value and hence the amplitude of THz radiation. By changing magnetic field direction, polarization plane of THz wave can be controlled^16^.

New way for THz emission polarization control was demonstrated in Ref.^17^. In a Co/Pt heterostructure with in-plane magnetization of Co layer, not only a linearly polarized, but also a circularly polarized femtosecond laser pulse launches a spin-current from Co to Pt. In the Pt-layer, the spin current is subsequently converted into a charge current due to the ISHE. For circularly polarized optical pulse used for excitation, the THz emitted pulse is linearly polarized, with polarization plane being helicity dependent. Polarization control of THz wave by spatially varying magnetic field (quadrupole-like in a demonstrator^18^) was suggested in Ref.^16^. Similar approach was used in THz near-field microscope^13^.

Spin-reorientation transition (SRT) is known as a bright example of the order-order type phase transitions in magnets^19,20^. These transitions appear as a change in orientation of magnetic moment under variation of the external parameters such as temperature, magnetic field strength, stress, etc. At the critical point of the first order SRT, the spin orientation changes abruptly. In the second order SRT, orientation changes continuously, but near the critical point the susceptibility of the spin system increases anomalously.

The effects induced near SRT by ultrafast optical excitation attract special attention. It was reported an enhancement of the spin precession amplitude and of the intensity of THz emission excited by femtosecond pulse in the vicinity of SRT temperature^21–23^. The latter effect provides a THz emitter with new mechanism, which is mostly attractive and economically viable if operates at room temperature. In most cases (Terbium–Yttrium Iron Garnets, Intermetallic Compounds RFe_2_, DyFeO_3_ type orthoferrites, etc.) the SRT occurs at low temperature. Some special materials like heterostructures^24–27^ or very thin films^28^ have been developed which exhibit SRT at room and even higher temperatures. So far, the light induced effects have been demonstrated only near SRT induced by temperature.

The first observation of the spin dynamics excited by femtosecond laser pulse in the vicinity of SRT induced at room temperature by magnetic field has been reported in Refs.^29,30^. The experiments were carried out on the intermetallic TbCo_2_/FeCo heterostructure with uniaxial in-plane magnetic anisotropy.

In this paper, we report on THz emission in the TbCo_2_/FeCo heterostructure. The experimental method makes it possible to indicate a most probable mechanism of the emission as optically induced ISHE. ISHE in this structure was observed previously using direct spin current injection from YIG film at ferromagnetic resonance^31^. In the area of SRT we demonstrate manipulation of THz wave polarization by variation of magnitude of the DC magnetic field while its direction is fixed. Manipulation is made without losing the generated total THz field. Intriguingly, the whole scenario unfolds for the magnetic field strength below the SRT critical value.

## Results

### THz signal hysteresis

THz emission was generated by linearly polarized femtosecond laser pulse incident normally on a multilayer TbCo_2_/FeCo structure with in-plane magnetization. A detailed sample description and the schematic of the optical experiment are presented in the “Method” section.

**Figure 1:**
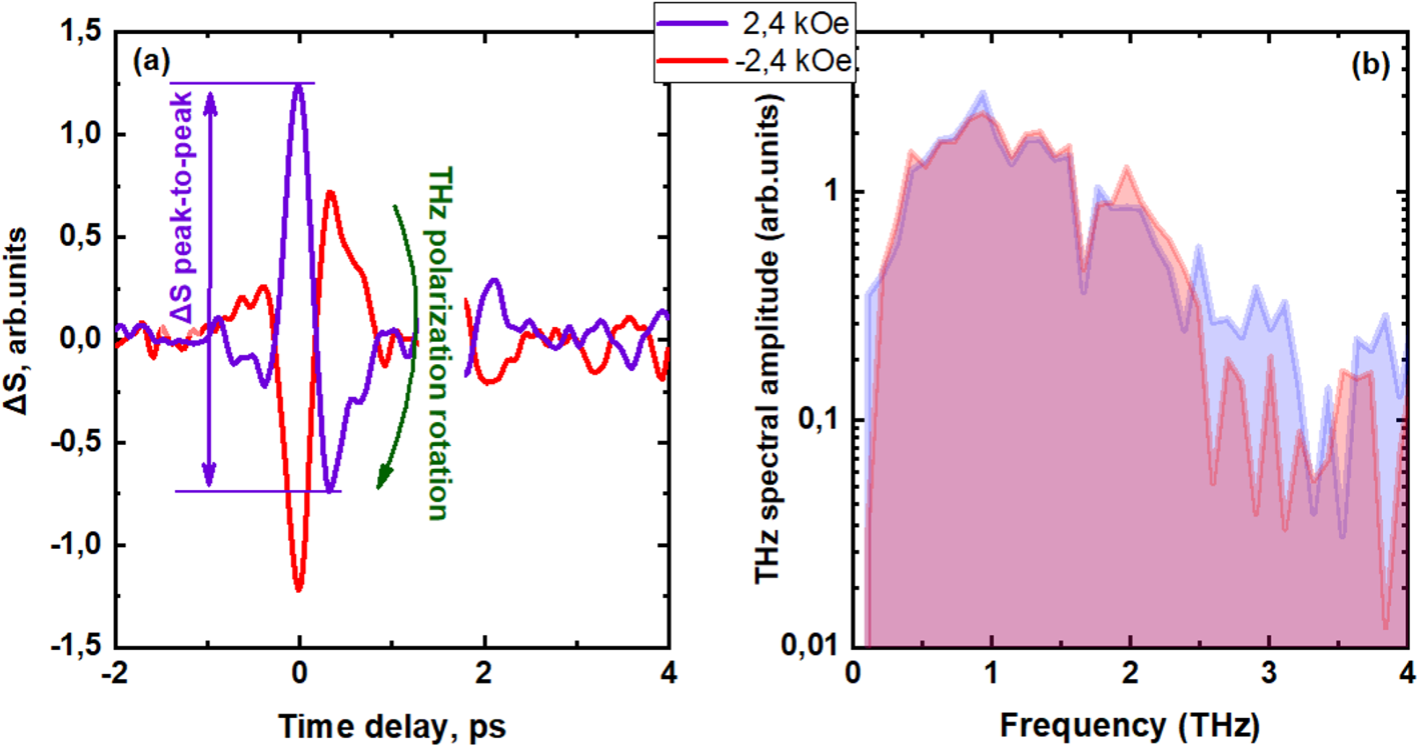
(**a**) Time-domain profile of the THz signal generated in the “hard axis” geometry. Green arrow shows the change in the THz polarization angle by 180 degrees. Red arrow shows the peak-to-peak amplitude; (**b**) correspondent emission spectra.

The emission was studied for two characteristic orientations of magnetizing field relatively to the axis of the uniaxial magnetic anisotropy of the structure: the magnetic field vector either parallel to the easy axis (“easy axis” geometry) or normal to it (“hard axis” geometry). Time domain profile of the THz signal Δ*S* in the “hard axis” geometry is shown in Fig. 1a and appropriate spectra is shown in Fig. 1b (for exact definition of Δ*S* see Supplementary Information S5). A change in the sign of the magnetizing field inverts the THz pulse, that is reversing the phase of the pulse.

**Figure 2:**
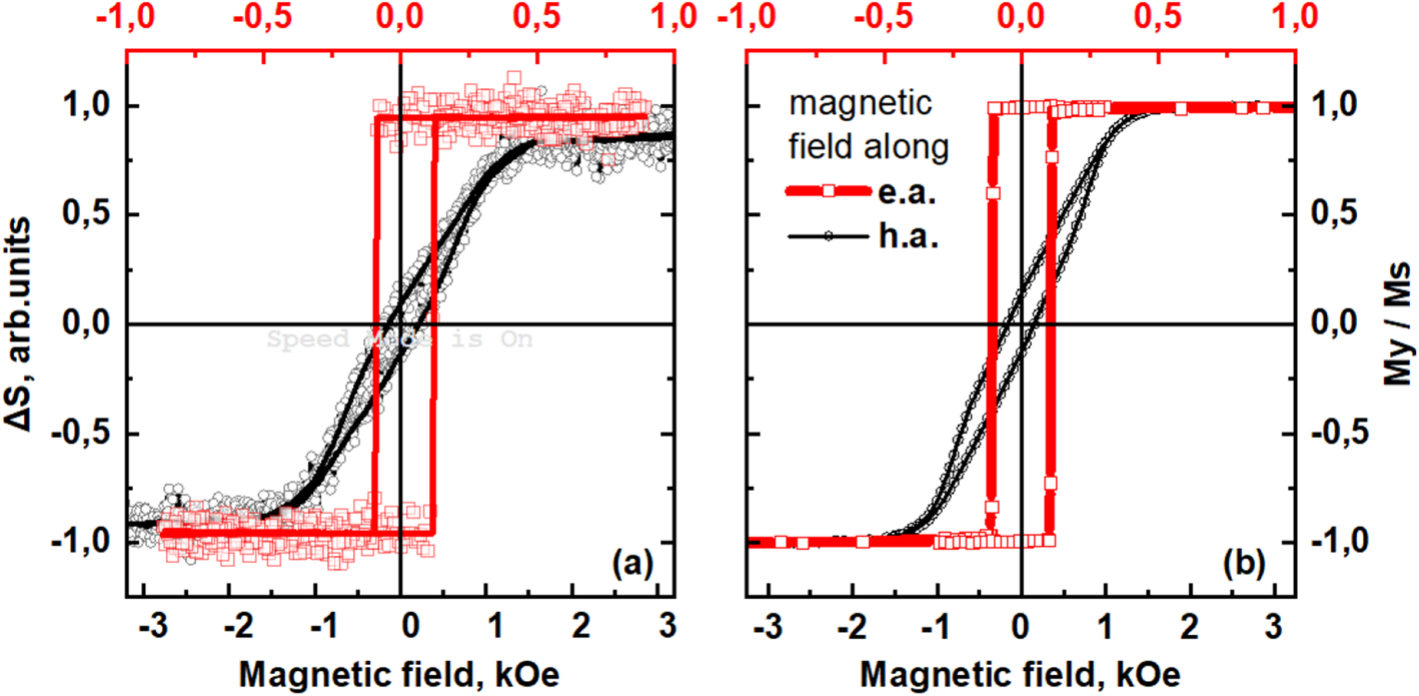
Comparison of the hysteresis loops of magnetization and change in THz signal depending on the applied magnetic field. (**a**) Hysteresis loops of the THz signal $\Delta S$, measured stroboscopically at $t = 0$ ps (which corresponds to the maximum signal in the time domain profile) with magnetic field applied in the “easy axis” (e.a.) and “hard axis” (h.a.) geometries; (**b**) the corresponding magnetization hysteresis loops for ${M}_{y}\left(H\right)$. Note the difference for top and bottom scales.

The dependences of the maximum of the THz signal $\Delta S$ (that is at $t = 0$) on magnetic field strength in the “easy axis” and “hard axis” geometries (THz hysteresis loops) are presented in Fig. 2a. The obtained dependences coincide with the corresponding magnetization curves (Fig. 2b) measured by VSM technique. In the “easy axis” geometry, the THz signal possesses almost rectangular hysteresis loop with the coercive field about 0.1 kOe. In the “hard axis” geometry, the field dependence of the THz signal reproduces magnetization curve typical for SRT. The saturation along magnetic field occurs at about 1,2 kOe when magnetizing field achieves the value of the anisotropy field ${H}_{A}$. The field strengths $H=\pm {H}_{A}$ correspond to the critical points of the SRT. A decrease in the field strength is accompanied by a tilt of magnetic moment towards the “easy axis”, which represents a transition from saturation to the angular (canted) magnetic phase. In the interval − ${{H}_{A}}<H<+{{H}_{A}}$, the magnetic moment gradually rotates from negative to positive orientation, and its projection onto the "hard axis" linearly depends on $H$ value. The amplitude of the emission does not depend on magnetic field when magnetic states are saturated along $H$.

### THz polarization rotation

**Figure 3:**
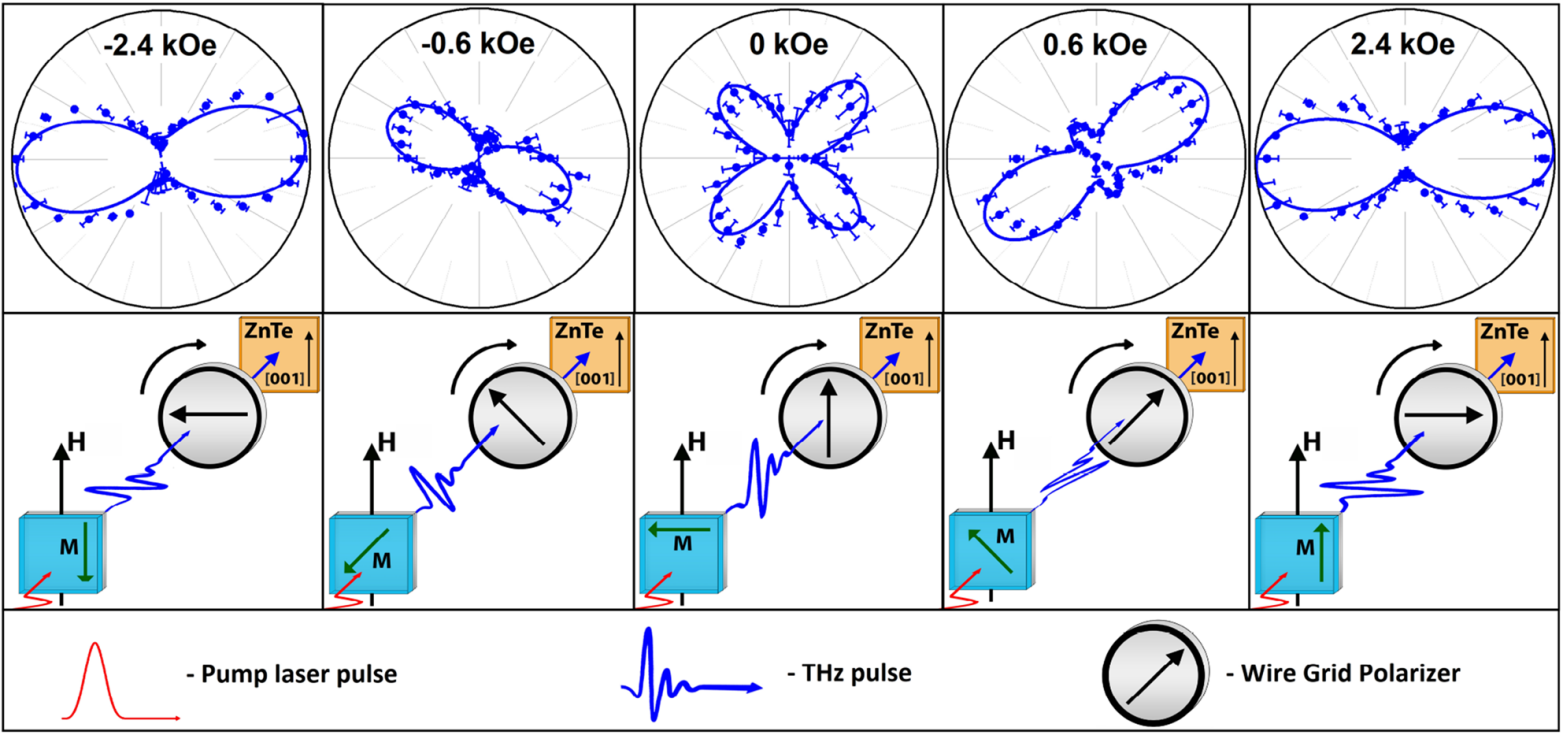
Angular diagram (peak-to-peak THz amplitude as a function of ${\varphi }_{2}$) for different orientations of the wire grid polarizer and different values of magnetic field in the “hard axis” geometry. The scale is the same for all polar dependences. Lines are fits to data using by Eq. (S4) from Supporting Information file. Sketch on bottom shows corresponding directions of THz wave polarization. The direction of magnetization is shown on the sample with a green arrow and denoted by the letter $M$. The direction of application of the magnetic field is denoted as $H$. The THz field is always perpendicular to the magnetization $M$.

To study the effect of the magnetic field strength on the polarization of generated THz pulses, a THz polarizer was installed in the path between the ZnTe crystal detector and the sample. The principle of polarization determination by rotation of the polarizer when optical axis of the detector is fixed is described in the “Method” section. The experimental dependences of the THz signal peak-to-peak amplitude (as shown in Fig. 1a) on the angle of rotation of the polarizer in the “hard axis” geometry are shown in Fig. 3. The dependences reflect THz polarization rotation during spin reorientation controlled by magnetic field strength in the SRT area.

**Figure 4:**
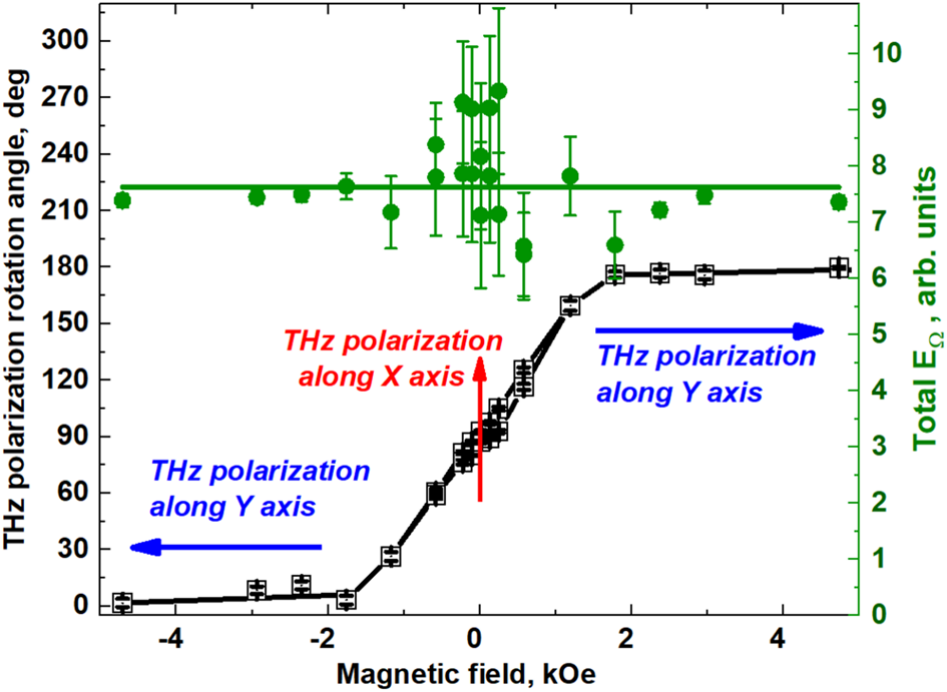
The dependence of the polarization angle and the magnitude of the THz field at different values of the magnetic field. Left scale: Magnetic field dependence of the THz polarization angle obtained from the fits of angular diagrams (Fig. 3) in the “hard axis” geometry. Right scale: total THz field dependence as fitting parameters from Eq. (S4) from Supporting Information file. Lines are guides for eye.

Figure 4 shows the THz polarization angle as a function of the magnetic field strength measured stroboscopically in the “hard axis” geometry (see the “Method” section). As can be seen, the result completely matches the field dependence of the THz signal shown in Fig. 2a.

**Figure 5:**
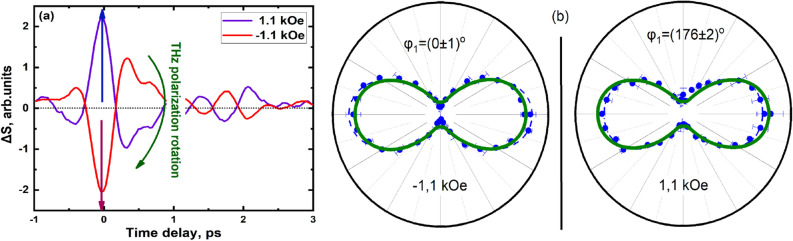
(**a**) Time-domain profile of the THz signal generated in the “easy axis” geometry; (**b**) angular diagram of the THz signal detected at variable orientations of the polarizer for different two values of the magnetic field in the “easy axis” geometry. Blue dashed lines are fits to data by Eq. (S4) with ${\varphi }_{1}$ as fitting parameter. By the olive lines, the “ideal” diagrams for 180° rotation are shown.

Similar studies were carried out in the “easy axis” geometry, the results are presented in Fig. 5. A sharp change in the phase of generated THz pulses did not allow for measurements in intermediate states between positive and negative magnetic phases in the given geometry. In this case, a phase change means a 180-degree THz polarization rotation relative to the X laboratory frame.

## Model and discussion

**Figure 6:**
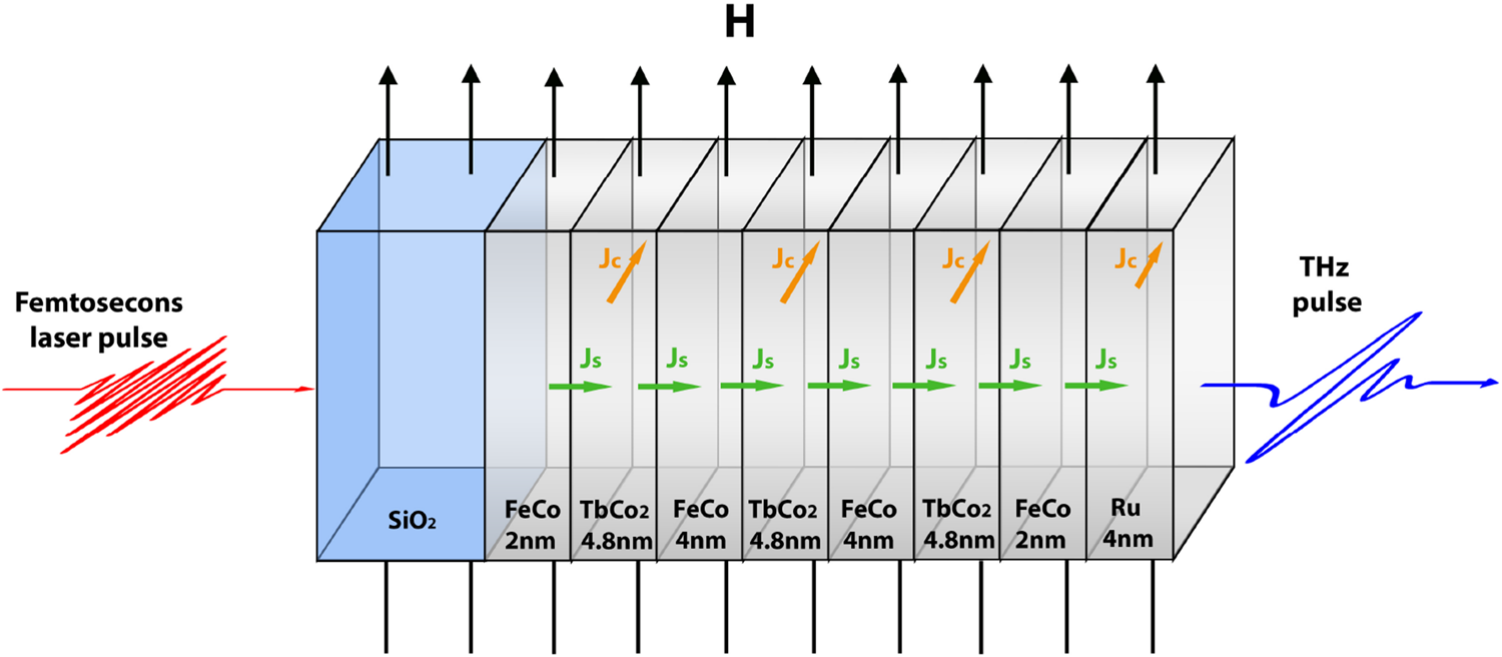
Illustration of the sample structure and directions of valuable vectors. $H$ is magnetic field strength, ${J}_{S}$ is the spin-current, ${J}_{C}$ is the charge current.

The observed effect of magnetic field on the amplitude of generated THz pulses in the TbCo_2_/FeCo heterostructure reveals ISHE mechanism of the THz emission similar to that described in Refs.^15,32^. Light-induced demagnetization in the FeCo layer launches a pulse of spin current into the TbCo_2_ and Ru layers (see Fig. 6). In the framework of ultrafast demagnetization mechanism, the direction of spin current is determined by asymmetric position of the Ru cap and by the gradient of magnetization quenching induced by laser irradiation. The spin current is normal to the structure surface $\overrightarrow{{J}_{s}}||\overrightarrow{z}$*.* Spin polarization the majority of electrons is antiparallel to magnetization $\overrightarrow{\sigma }||-\overrightarrow{M}$.

Strong spin–orbit interaction in TbCo_2_ splits electron flows with opposite spins, generating a charge current ${\overrightarrow{J}}_{c}$, according to ISHE spin-charge conversion:

$${\overrightarrow{J}}_{c}={\theta }_{ISHE}\left[ \overrightarrow{{J}_{s}}\times \overrightarrow{\sigma }\right]\propto \left[\overrightarrow{M}\times \overrightarrow{z}\right]$$

where ${\theta }_{ISHE}$ is electron deflection angle.

**Figure 7:**
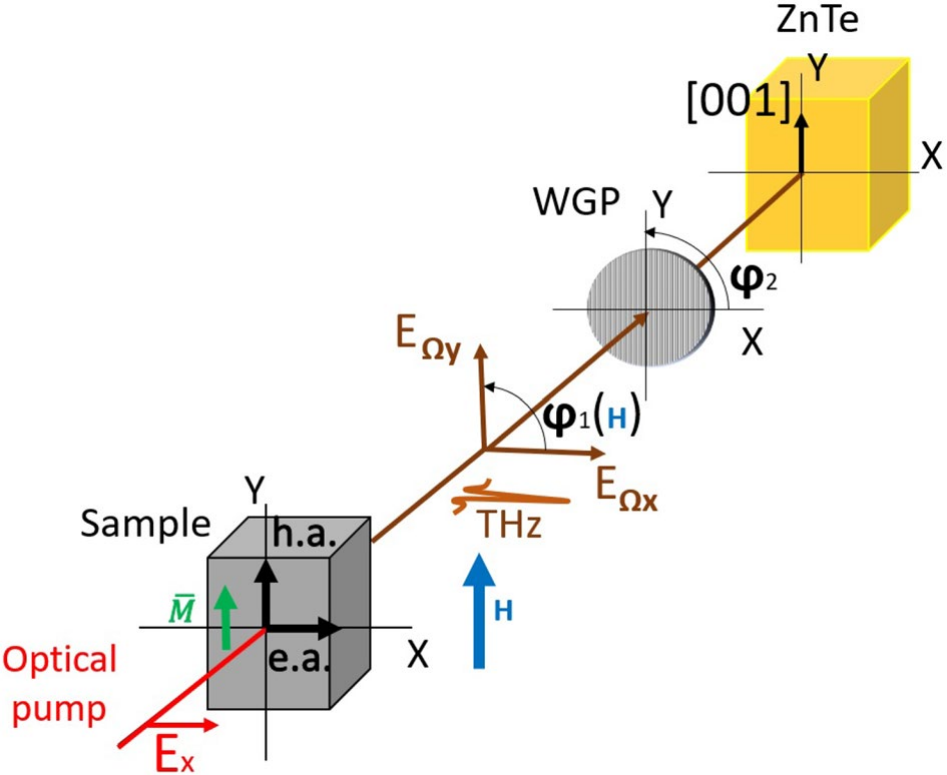
Schematic of the mutual orientation of the magnetic field, ZnTe [001] crystallographic axis, and polarization planes of optical and THz waves in “hard axis” geometry. XYZ—laboratory frame, ${\varphi }_{1}$ is the angle of rotation of THz wave polarization plane, ${\varphi }_{2}$ is the angle of rotation of the wire-grid polarizer (WGP).

The basic scheme of measurements (presented in Fig. 7 of the “Method” section) detects ${E}_{\mathrm{x}}$—component of THz electric field, that is normal to the optical axis [001] of ZnTe nonlinear crystal. Since polarization of the emitted wave is parallel to ${\overrightarrow{J}}_{c},$ the measured amplitude of THz pulses is proportional to the *Y*-component of magnetization: ${E}_{\mathrm{x}}\propto {M}_{y}$_._ In the scheme given in Fig. 7, the magnetizing field is parallel to *y*-axis, for this reason THz amplitude dependences on magnetic field reproduce magnetization curves ${M}_{y}\left(H\right)$ in both the “easy axis” and in the “hard axis” geometries. According to the ISHE mechanism, THz polarization direction should follow variations of magnetic moment orientation. In the angular (canted) phase of SRT, the orientation of the magnetic moment is controlled by the strength of magnetic field directed normally to the easy axis^20^.

The SRT in a uniaxial magnetic film arises in the canted state as pure rotation of saturated magnetization by 90° from hard to easy axis when magnetic field value $H$ decreases from ${H}_{A}$ to 0. After changing the sign of the magnetic field, the magnetization continues to rotate up to 180° when negative value of $H$ decreases down to − ${H}_{A}$. In the framework of ISHE mechanism such a pure rotation of magnetization is accompanied only by the change of the current direction without changes of its value. Thus, THz polarization can rotate at fixed intensity of the emission. This opens a way for control of THz polarization without rotation of the magnetic field.

## Conclusions

We presented the results of a study of THz emission generated by femtosecond laser pulses in the spintronic TbCo_2_/FeCo heterostructure. The THz pulses generated in these structures show linear polarization. The total intensity of THz emission depends only on the optical pump intensity and does not depend on the magnetic field. Magnetic field provides control over polarization of THz wave by its strength: a fairly small field within the range ${H}_{A}$ rotates polarization within entire angular range of ± 180°. For our samples ${H}_{A}$ = 1.2 kOe and can be significantly reduced for the specially fabricated samples^25^. Continuous polarization control can be achieved for the “hard axis” configuration, i.e. when magnetic field is applied perpendicularly to the magnetization axis of the sample. In the “easy axis” configuration, changing magnetic field results only in reversing the phase of the THz pulse.

The suggested way of polarization control is based on the magnetic-field induced spin-reorientation phase transitions in the TbCo_2_/FeCo heterostructure. The SRT provides magnetization rotation when magnetic field is changed by its value only, giving rise to polarization rotation of the THz pulse. This makes the SRT multilayers very different from the magnetic/nonmagnetic structures. In the latter, the polarization rotation can be achieved by rotation magnetic field around them, that is much less technologically justified.

It is worth noting that in the studied magnet/magnet structures, THz generation is provided by inversed spin-Hall effect, analogously to that in the commonly used magnet/nonmagnet structures. However, in our work we show that this mechanism can be generalized to a magnet/magnet multilayer structure as well.

Spin reorientation phase transitions that have proven to be the basis for creating innovative spintronic and straintronic functional materials and devices are applied here to generation of controllable THz emission. The proposed THz polarization control by SRT is applicable independently on mechanism of the THz emission proved that polarization direction is determined by the magnetic moment orientation.

The results obtained allow us to consider SRT as a promising approach for elaboration of THz emitters with controllable parameters.

## Method

### The sample

The multilayer film FeCo 2 nm/TbCo_2_ 4.8 nm/FeCo 4 nm/TbCo_2_ 4.8 nm/FeCo 4 nm/TbCo_2_ 4.8 nm/FeCo 2 nm/Ru 4 nm was deposited on a glass substrate by RF sputtering technology (Fig. 6). Ruthenium is non-magnetic cap layer. The sputtering was carried out under the static magnetic field that allows for fabrication of heterostructure with controllable uniaxial in-plane magnetic anisotropy^25^.

The sample was mounted on a non-magnetic holder between two electromagnet poles, which allowed applying fields up to 5 kOe in the plane of the sample along Y axis of the laboratory frame (Fig. 7). A GMW electromagnet (Model 3470) was used as the source of the magnetic field.

Rotation of the sample around the normal to its surface made it possible to set the sample with the easy axis of magnetization directed either parallel or perpendicular to the magnetic field: “easy axis” geometry (e.a.) or “hard axis” geometry (h.a.), respectively.

### Optical geometry

The THz emission characteristics were studied using a time-domain spectroscopy scheme, for details see Supplementary information (SI).

For pumping, an amplified Titanium:Sapphire based femtosecond laser system (TiF-20F, Avesta project, Russia) was used provided a 35 fs laser pulse with a repetition rate of 3 kHz at a central wavelength of 800 nm. The output optical radiation was split into two beams: an optical pump pulse and an optical probe pulse. An optical pump pulse was focused onto the sample surface, the energy density being ~ 1 mJ/cm^2^. The generated in the sample THz radiation was collimated by a parabolic mirror into a parallel beam and then, by the second parabolic mirror, was focused onto the surface of the ZnTe crystal detector. The optical pump radiation was cut off by a filter. The probing beam, passing through the delay line, was focused onto the surface of the ZnTe crystal detector as well and was spatially overlapped with the THz pulse. After interacting with the detector crystal, the probe beam passed through a polarizer, the optical plane of which was crossed with the probe beam polarization plane (zero-point electro optical regime measurements^33^).

To study the polarization characteristics of the generated THz radiation, a wire-grid polarizer (WGP) was used, which could be rotated through an angle ${\varphi }_{2}$ (For geometry, see Fig. 7). The polarization of the pump and probe beams was rigidly fixed, so that the pump polarization was oriented horizontally in the laboratory axis X, and the polarization of the probe beam was vertical in the laboratory axis Y. In accordance with Ref.^34^, the polarization of the probe beam was set parallel to the [001] axis of the ZnTe detector crystal, which allows to detect the X-component of the THz field (${E}_{\mathrm{x}}$). For details of the method for determining the direction of the emitted THz pulse polarization can be found in SI.

### Optical measurements

The following types of measurements have been performed.

1. Time-domain profiles of THz pulses generated by the spintronic structures in a pump-probe configuration (by changing the delay time between the pump and probe pulses with and without a magnetic field application): Fig. 1a.
2. Stroboscopic measurements of the THz pulse amplitude at the fixed delay time during magnetic field sweep (THz amplitude hysteresis): Fig. 2a.
3. Peak-to-peak measurements are the result of hardware and software processing of time domain dependences, measured as in item 1) at different values of magnetic field (see also SI). For each $H$, peak-to-peak value (see Fig. 1a) was extracted.
4. THz emission angular measurements. Peak-to peak measurements were performed with a chosen position ${\varphi }_{2}$ of WGP; ${\varphi }_{2}$ was varied from 0° to 360° with 10°—step. In this way, angular dependences as in Figs. 3 and 5 were obtained.
5. THz pulse polarization angle ${\varphi }_{1}$ determination: as a fit of angular efficiency measurements (from Figs. 3, 5, the result is shown in Fig. 4).

All experiments were carried out at room temperature.

## Supplementary Information

Supplementary Information.
