# Supplementary material for: Polarization control of THz emission using spin-reorientation transition in spintronic heterostructure
Source: Sci Rep. 2021 Jan 12;11:697. doi: 10.1038/s41598-020-80781-5 (PMC7804947; doi:10.1038/s41598-020-80781-5)
Supplement: Supplementary file 1 — Supplementary Information. [file 41598_2020_80781_MOESM1_ESM.pdf]

Supplementary Information

POLARIZATION CONTROL OF THz EMISSION USING SPIN-REORIENTATION TRANSITION IN SPINTRONIC HETEROSTRUCTURE

Dinar Khusyainov[1], Sergei Ovcharenko[1], Mikhail Gaponov[1*], Arseniy Buryakov[1], Alexey Klimov[1], Nicolas Tiercelin[2], Philippe Pernod[2], Vadim Nozdrin[3], Elena Mishina[1], Alexander Sigov[1] and Vladimir Preobrazhensky[3]

[1] MIREA - Russian Technological University, Moscow, 119454, Russia
[2] Univ. Lille, CNRS, Centrale Lille, ISEN, Univ. Valenciennes, UMR 8520 - IEMN, Lille, F-59000, France
[3] Prokhorov General Physics Institute of RAS, Moscow, 119991, Russia

* mikhail.lab109@gmail.com

Supplementary Note 1: THz generation efficiency as function of pump pulse fluence

To determine the efficiency of THz radiation generation in the $TbCo_2$/FeCo structure, the ΔS peak signal (Supplementary S5) was measured at different values of the incident radiation power (Supplementary Figure 1). Detected signal, which is proportional to E_{Ω}^2 [1], nonlinearly depends on the pump fluence due to nonlinearity of inverse spine Hall effect [2].

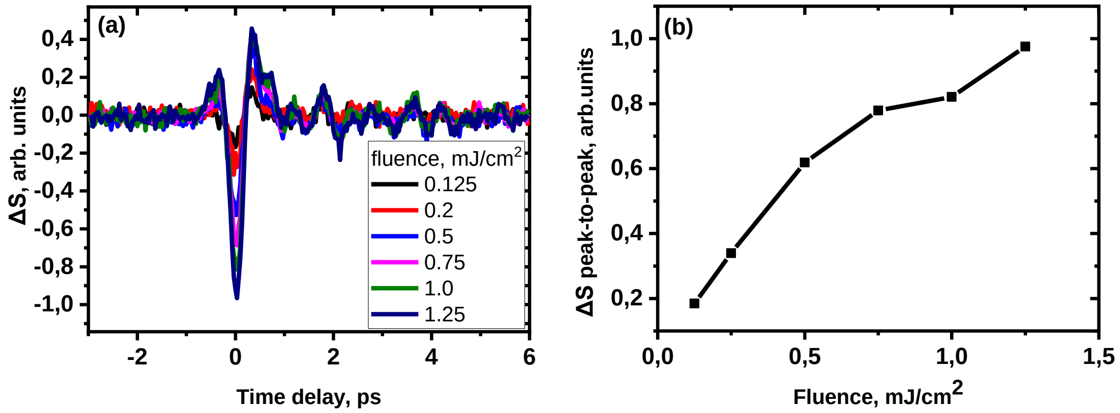

Supplementary Figure 1: (a) – Time domain profiles of the ΔS signal induced by optical pump pulse of different fluence; (b) – Dependence of the ΔS peak-to-peak values on the optical pump pulse fluence. Line in (b) is guide to eye.

Supplementary Note 2: THz generation efficiency as function of pump polarization

Efficiency of THz emission was checked as function of the optical pump polarization (Supplementary Figure 2). It was found that the ΔS peak-to-peak signal remains constant for any direction of the pump polarization.

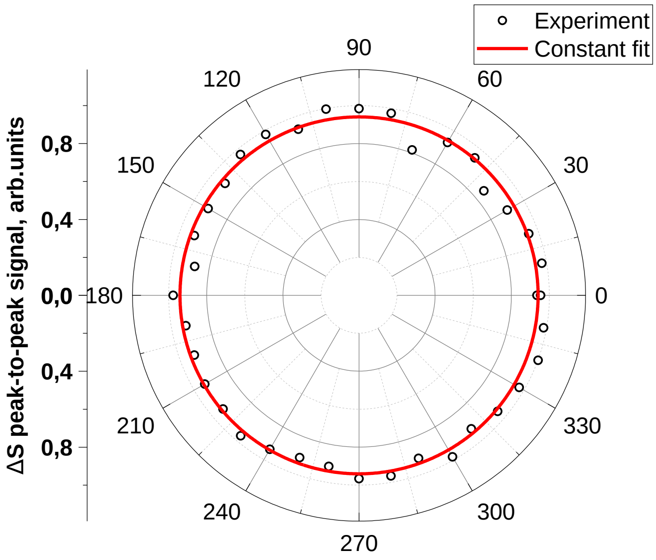

Supplementary Figure 2: Dependence of the ΔS peak-to-peak signal on the polarization of the pump optical pulse.

Supplementary Note 3: Experimental Schematic

To determine the magnitude and direction of the polarization of THz radiation generated by the investigated sample and its dependence on the magnetic field, the method of electro-optical sampling was used [3]. The experimental setup is shown in Supplementary Figure 3.

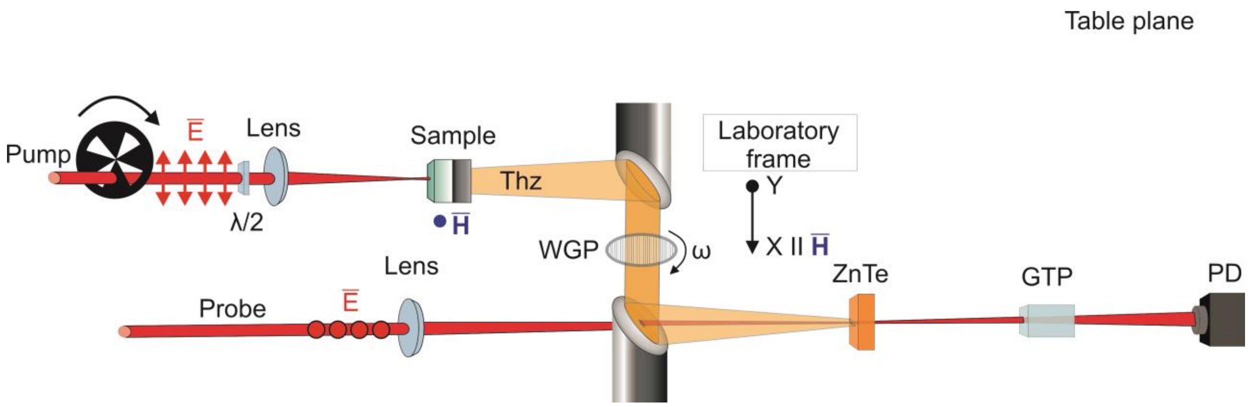

Supplementary Figure 3. Schematic representation of the set-up, where WGP is wire-grid polarizer, GTP is Glan Taylor polarizer, PD is photodiode

This method is based on the linear electrooptic Pockels effect. We used the scheme suggested in [3] with ZnTe as an electro-optical detector crystal. The crystal is cut perpendicular to the crystallographic axis [110] (i.e. in (110) plane, see Supplementary Figure 4). A linearly polarized optical pulse (probe) propagates coaxially with a terahertz pulse and falls normally onto the surface of the ZnTe crystal. Therefore, both in the optical probe and in THz pulses, the electric field vectors lie in the (110) plane of the crystal.

A polarizer (WGP) is used to analyze the THz polarization generated in the sample. As a result of interaction of THz and input optical pulses in ZnTe, the polarization of the probe laser pulse becomes elliptical. The final intensity of the probe passing through the ZnTe depends on:

1) the initial orientation of the probe polarization relative to the anisotropic crystal axis;
2) both the magnitude and orientation of electric field of the THz pulse (see Supplementary Figure 4);
3) the thickness of the crystal, which determines the phase shift between ordinary and extraordinary waves;

Supplementary Note 4: Calculation of detected X-component of THz field

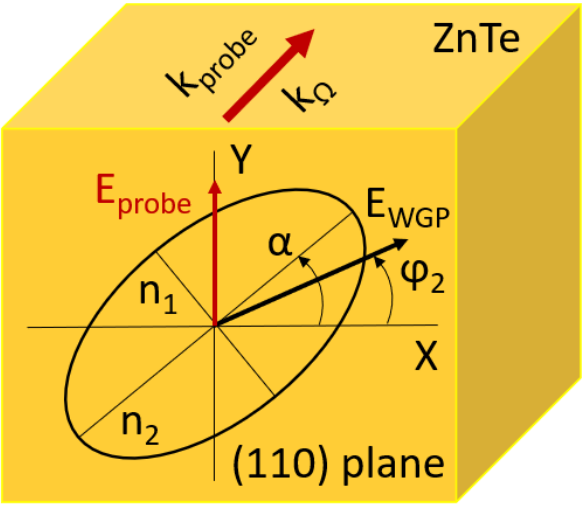

Supplementary Figure 4: Orientation of ZnTe in the experimental setup. Axis [001] and [-110] of the ZnTe crystal are parallel to the axes laboratory frame Y and X, respectively (see Supplementary Figure 3); E_{probe} — the electric field vector of the linearly polarized probe beam; E_{WGP} - orientation of linearly polarized THz electric field vector (after the WGP polarizer); n_1, n_2 are the refractive indices of an anisotropic crystal at $E_{WGP} \neq 0$, α is the orientation of the main axis of the ellipsoid.

In the absence of the THz field, the probe polarization coincides with the crystal axis [001] and the axis of the refractive index ellipsoid. As a result of this interaction, the polarization of the probe beam remains linear and does not change its orientation. This beam is blocked by the GTP. Electric field of THz pulse rotates the ellipsoid of the refractive indices and results in the ellipticity of the probe beam. The intensity of the signal detected by the diode is described by the following expression [1]:

$$S_{probe} = I_0 sin^2(2\,\alpha(\varphi_2)) sin^2\left(\frac{\Gamma(\varphi_2)}{2}\right) \quad \text{(S1)}$$

where I_0 is the probe beam intensity, $\alpha(\varphi_2)$ an angle of ellipsoid axis with the [-110] ZnTe axis on E_{WGP} orientation angle φ_2, Γ is the relative phase shift between the two orthogonal components of the laser field.

$$\Gamma(\varphi_2, E_\Omega) = \frac{\pi d}{\lambda_{probe}} n_{ZnTe}^3 r_{41} E_{WGP} \sqrt{1 + 3(cos\,\varphi_2)^2} \quad \text{(S2)}$$

where λ_{probe} is the wavelength of the probe wave, d is the ZnTe thickness, n_{ZnTe} is the ZnTe refractive index for λ_{probe}, r_{41} is the electro-optical coefficient, E_{WGP} is the field amplitude at the output of the THz polarizer. The amplitude of the THz field depends on the mutual polarization orientation of the THz field and the WGP axis:

$$E_{WGP} = E_\Omega \cos(\varphi_2 - \varphi_1) \quad \text{(S3)}$$

So, the resulting intensity of the probe beam measured by the diode is given by:

$$S_{probe}\,(\varphi_2, E_\Omega) = I_0\, sin^2(2\,\alpha(\varphi_2))\, sin^2\left(\frac{\frac{\pi d}{\lambda_{probe}} n_{ZnTe}^3 r_{41} E_\Omega \cos(\varphi_2-\varphi_1)\sqrt{1+3(\cos\varphi_2)^2}}{2}\right) \approx$$

$$\approx \left\{ I_0\, sin^2(2\,\alpha(\varphi_2))\left(1.9 \cdot 10^{-7} \cdot \cos(\varphi_2 - \varphi_1)\sqrt{1 + 3(\cos\varphi_2)^2}\right)^2 \right\} (E_\Omega)^2 \quad \text{(S4)}$$

where the smallness of the argument under sine is taken into account.

In order to increase the signal-to-noise ratio, the final signal recorded by a synchronous detector (Lock-in amplifier SR830) is given by:

$$\Delta S(t) = S_{probe}(\varphi_2, E_\Omega(t)) * Cos(\Theta(t)) \quad \text{(S5)}$$

where Θ is the phase difference between $\Delta S(t)$ and the reference signal, t is the delay time [3].

SUPPLEMENTARY NOTE 5: Configuration of E_{Ω} and WGP in polarization detection experiment

If the THz polarization generated by the heterostructure is linearly polarized, then the following typical cases are probable (Supplementary Figure 5).

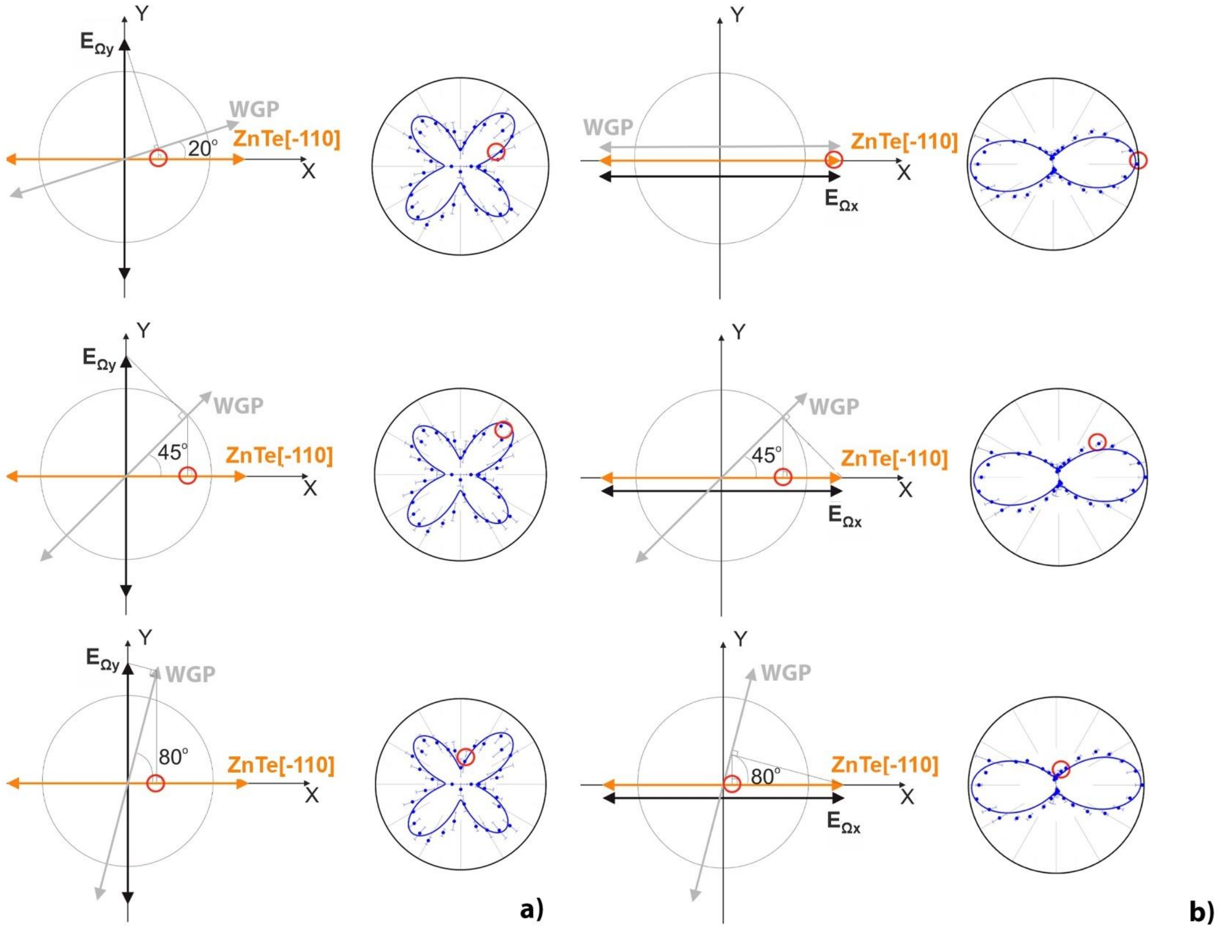

Supplementary Figure 5: Dependence of the signal recorded by the photodiode during WGP rotation for two characteristic orientations of the linearly polarized THz field: a) H = 0, E_{Ω}=$E_{\Omega y}$; b) H = 2.4 kOe, E_{Ω}=$E_{\Omega x}$

SUPPLEMENTARY REFERENCES

1. Brunken, M., et al. Electro-optic sampling at the TESLA test accelerator: experimental setup and first results. TESLA Report, **11** (2003).

2. Kampfrath, T., et al. Terahertz spin current pulses controlled by magnetic heterostructures. Nature nanotechnology **8**, 256 (2013).

3. Jiang, Z., et al. Electro-optic sampling near zero optical transmission point. Appl. Phys. Lett. **74**, 1191 (1999).
